# Preseptal and Postseptal Orbital Cellulitis of Odontogenic Origin

**DOI:** 10.7759/cureus.5087

**Published:** 2019-07-06

**Authors:** Tej G Stead, Armando Retana, Jessica Houck, Bryan C Sleigh, Latha Ganti

**Affiliations:** 1 Emergency Medicine, Brown University, Providence, USA; 2 Oral and Maxillofacial Surgery, Capital Center for Oral and Maxillofacial Surgery and for Cosmetic Surgery, Washington, USA; 3 Emergency Medicine, University of Central Florida College of Medicine / Hospital Corporation of America Graduate Medical Education (HCA GME) Consortium, Kissimmee, USA; 4 Emergency Medicine, Mercer University School of Medicine, Macon, USA; 5 Emergency Medicine, Envision Physician Services, Orlando, USA

**Keywords:** orbital cellulitis, preseptal cellulitis, odontogenic orbital cellulitis, periorbital cellulitis, postseptal cellulitis

## Abstract

The authors present a case of combined preseptal and postseptal cellulitis of odontogenic origin. The infection started as a dental abscess associated with a first maxillary molar. The infection spread into the paranasal sinus, developed into a pansinusitis, and then spread into the preseptal and postseptal tissues. In addition to extraction of the infected tooth, the patient underwent bilateral nasal endoscopy, maxillary antrostomy, total ethmoidectomy, sphenoidotomy, and frontal sinusotomy with balloon dilation. Sinus cultures were positive for 2+ microaerophilic streptococci.

## Introduction

Periorbital cellulitis, which is sometimes referred to as preseptal cellulitis, is an infection of the anterior portion of the eyelid [1]. In contrast, orbital cellulitis, which is also referred to as postseptal cellulitis, is an infection of the contents of the orbit (periorbital fat, extraocular muscles, and neurovascular bundles) [2]. Neither of these infections involve the globe itself. It is difficult but important to distinguish between preseptal and postseptal cellulitis as both of them can present with ocular pain and eyelid edema and erythema. Preseptal cellulitis is a less serious condition that rarely evolves into more serious complications. However, postseptal cellulitis can lead to vision loss as well as death in cases where the infection spreads into the cranial vault. This case illustrates two important points: 1) preseptal and postseptal cellulitis of the orbit can occur concurrently; 2) imaging can reveal the additional pathology of an underlying subperiosteal abscess.

## Case presentation

A 26-year-old African-American male presented to our emergency department (ED) complaining of right eye swelling and pain for one day. His past medical history included anxiety and asthma. His only medication was an occasional hydrocodone acetaminophen tablet as needed for chest pain associated with his anxiety. He had no known drug allergies, no prior surgeries, and denied drug abuse of any kind.

One week prior to presentation, he experienced tooth pain in the right maxillary region and felt an abscess forming in his gums adjacent to the tooth that was hurting. Subsequently, he experienced worsening pressure in his maxillary sinus and frontal sinus consistent with sinusitis for five days. The patient also endorsed worsening nausea and emesis for two days, and one day of worsening right periorbital edema and erythema. He reported that on the day of admission, he was vomiting in the bathroom, felt dizzy and fell on the floor but does not remember hitting anything on the way down. He denied insect bites. He denied fevers but endorsed night sweats and chills for five days, and blurry vision of the right eye for one day.

**Figure 1:**
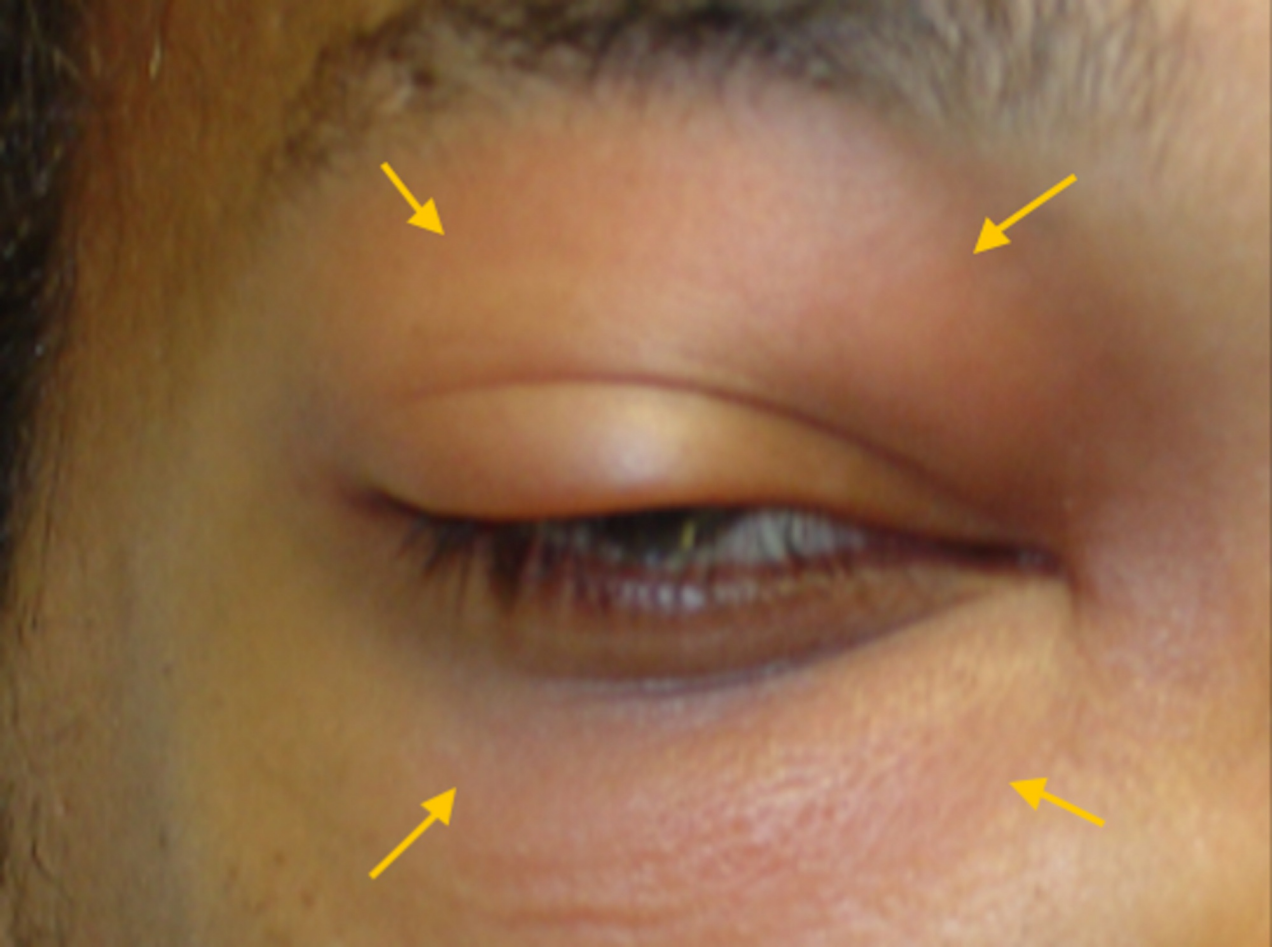
Clinical presentation of the patient. Note erythema, edema, and proptosis of the right eye (arrows).

On physical exam, his vital signs were stable and he was afebrile. The patient was sitting up in bed alert, awake, and oriented. He had significant right periorbital edema and erythema of the upper and lower eyelids with diffuse tenderness to palpation (Figure 1). Extraocular movements were intact, but he endorsed pain on medial and lateral gaze. He denied diplopia. Visual acuity in the right eye was 20/25 and 20/20 in the left eye. Pupils were equal, round and reactive to light. The nasal mucosa was erythematous but no nasal drainage was noted. An oral exam revealed multiple carious teeth with no associated fluctuant swelling or active draining fistulas, and his oropharynx was clear. The right maxillary canine was tender to percussion, but the tooth itself and adjacent teeth were vital and without gross decay. There was no cervical lymphadenopathy. His cranial nerve exam was within normal limits and the remainder of his physical exam was unremarkable.

All laboratory studies were unremarkable except for an elevated white blood cell (WBC) count of 22.7 * 10^9^ cells per liter of blood which were predominantly neutrophils, comprising 91.8% of the total.

**Figure 2:**
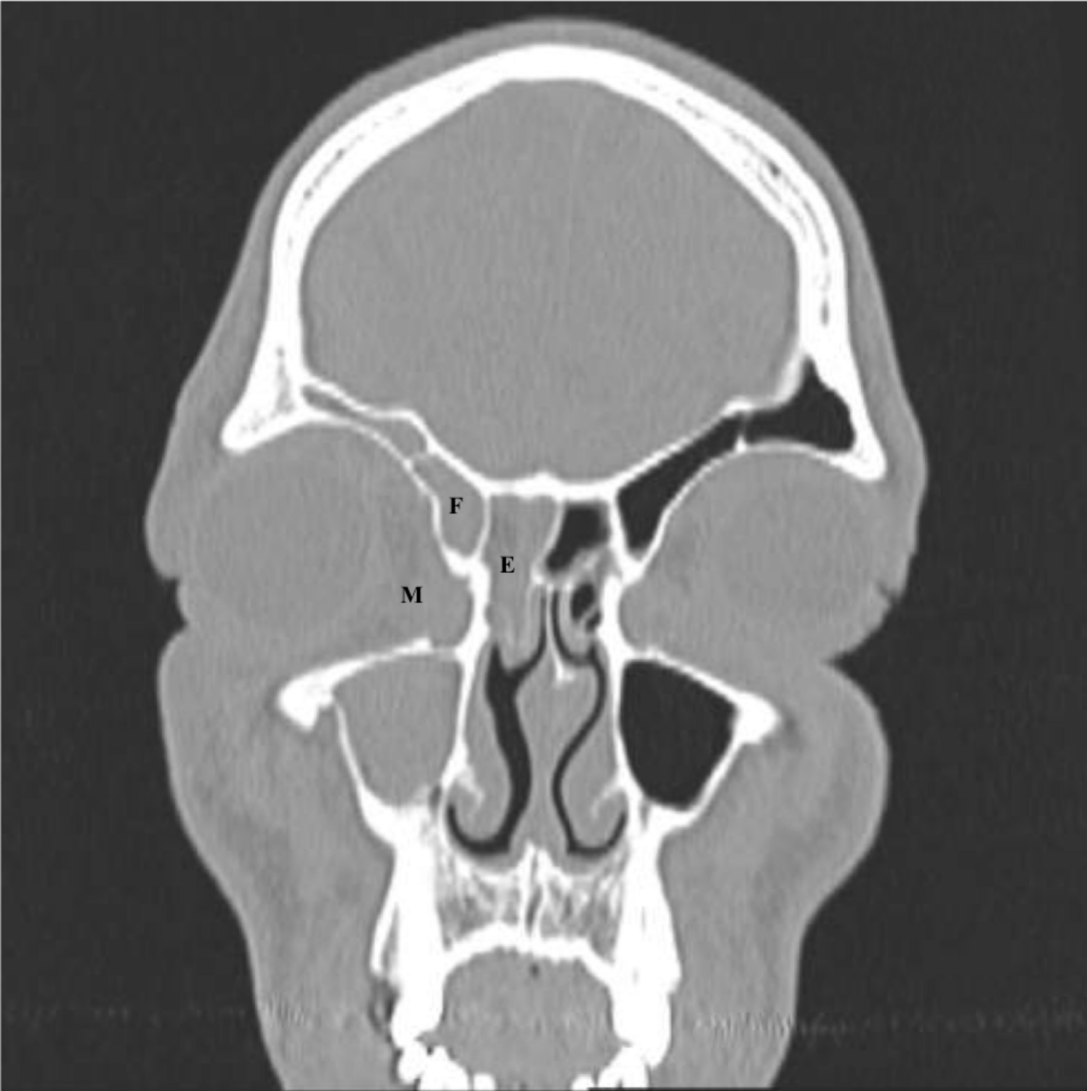
Opacification of the right maxillary, ethmoid, and frontal sinuses on coronal view of computed tomography of the head. M - maxillary, E - ethmoid, F - frontal.

**Figure 3:**
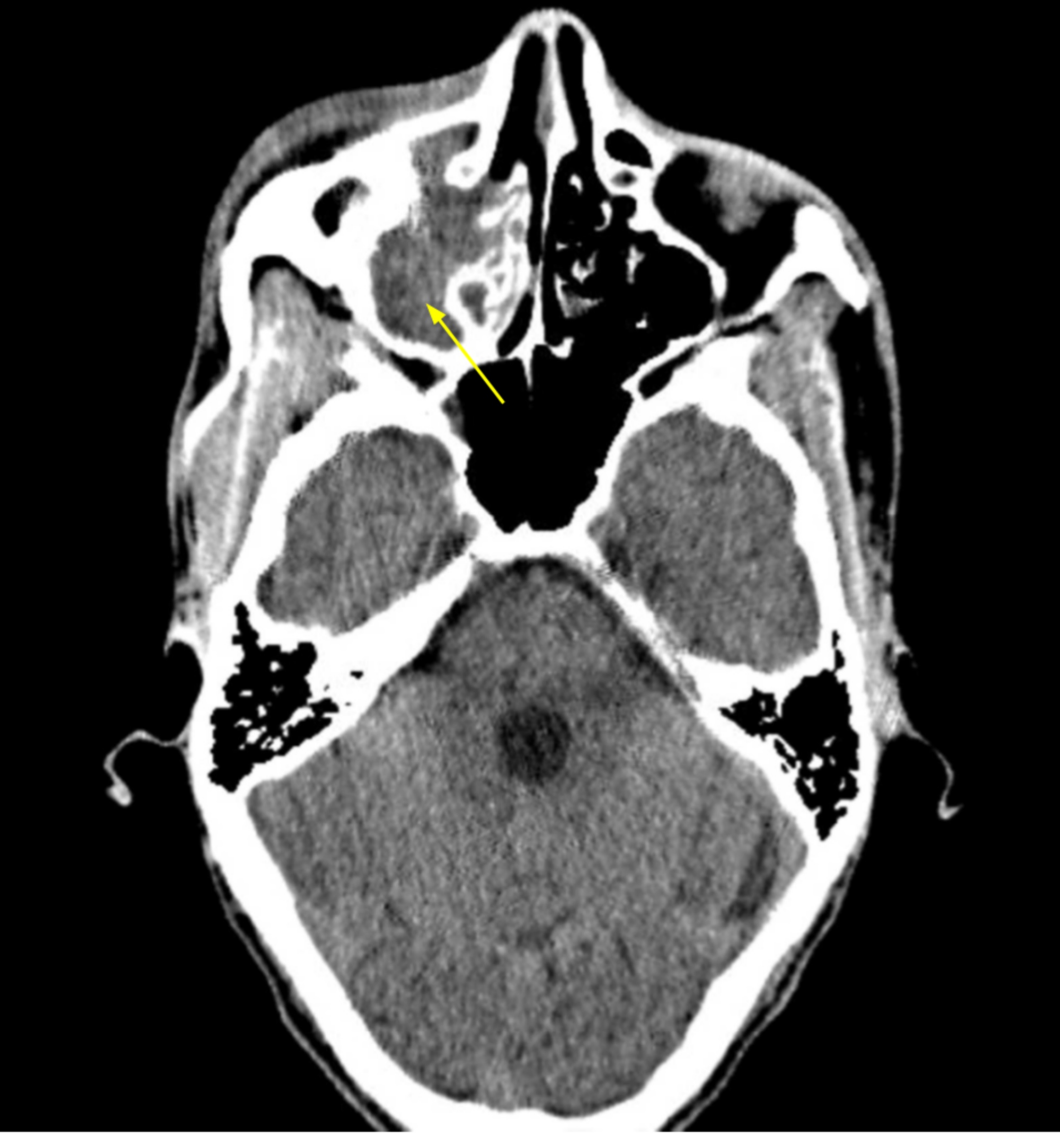
Opacification of the right maxillary sinus on computed tomography of the head.

**Figure 4:**
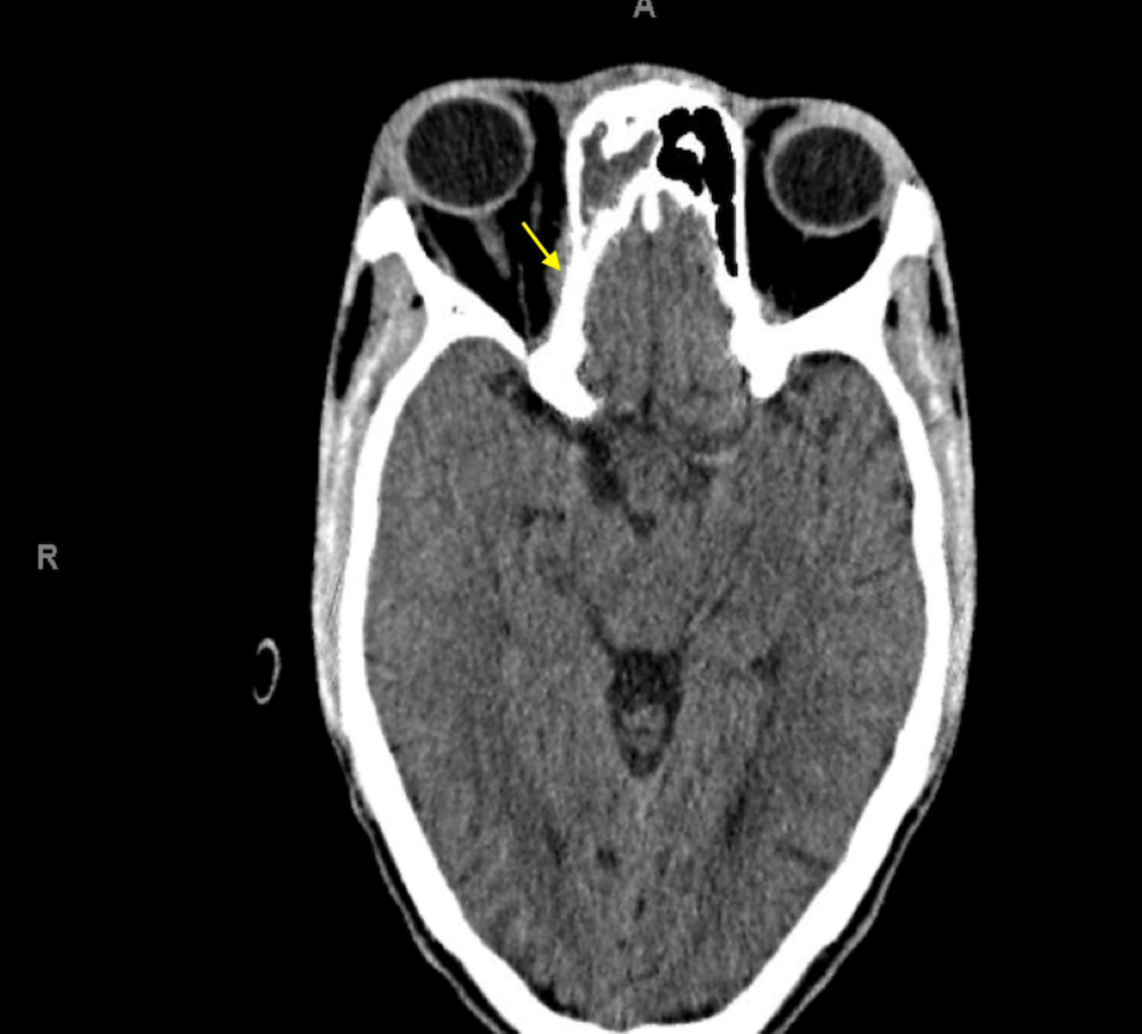
Opacification of the right ethmoid sinus on computed tomography of the head. Yellow arrow points to mild subperiosteal inflammation, which could be the beginning stages of a subperiosteal abscess.

**Figure 5:**
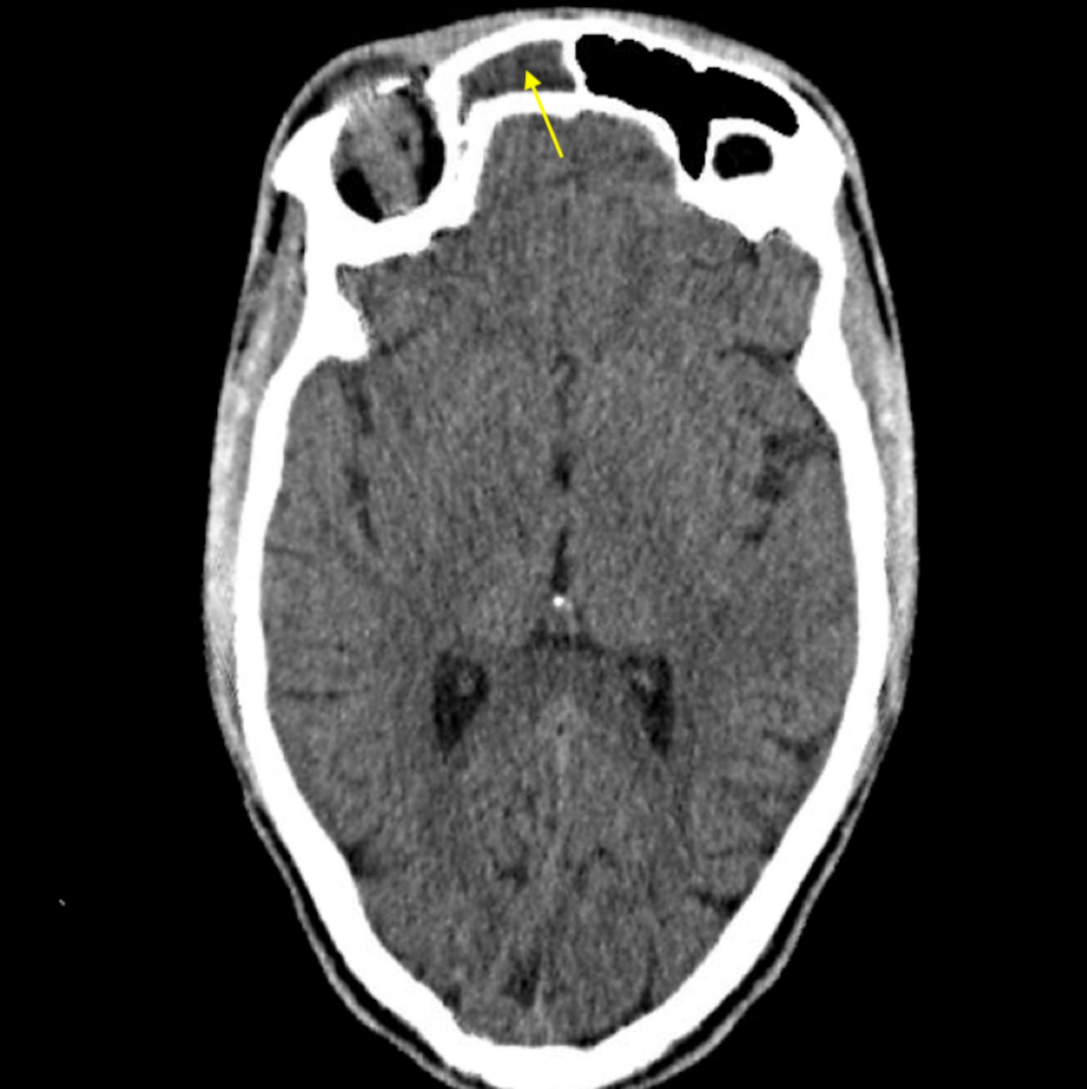
Opacification of the right frontal sinus on computed tomography of the head.

**Figure 6:**
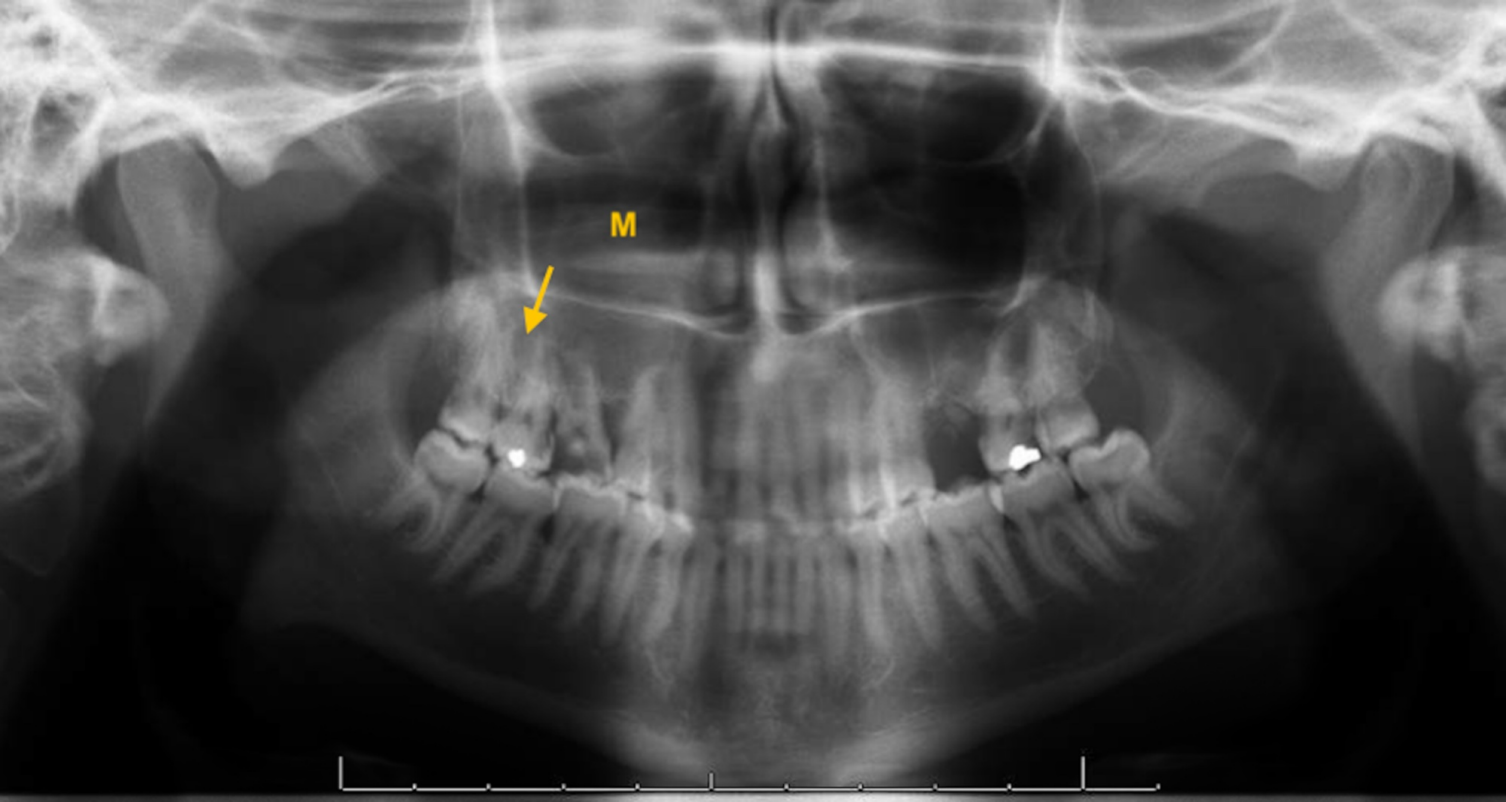
Tooth decay and periapical radiolucency (arrow) associated with maxillary right first molar with opacification of the right maxillary sinus (M) on the panoramic radiograph (orthopantomogram).

A non-contrast head computed tomography scan (CT) was ordered and revealed right globe proptosis with preseptal and postseptal soft tissue inflammation as well as full opacification of the right maxillary, ethmoid, and frontal sinuses (Figures 2-5). In addition, a subtle finding in this non-contrast study was noted on the orbital side of the right ethmoid bone, where one can observe a small soft tissue swelling which could be the beginning of a subperiosteal abscess. This finding could partly explain the proptosis of the right globe (Figure 5). There was no evidence of a cavernous sinus thrombosis, intracranial hemorrhage, mass, infarct or shift. Panoramic radiograph imaging revealed periapical radiolucency associated with maxillary right first molar, as well as tooth decay (Figure 6).

In the ED, he was given clindamycin 600mg intravenously (IV) and was admitted to the internal medicine team to continue treatment with IV antibiotics and for further work-up. The internal medicine team consulted oral and maxillofacial surgery (OMFS) for extraction of tooth #3, ophthalmology for evaluation of visual acuity and otorhinolaryngology (ENT) for opacification of paranasal sinuses. ENT took the patient to the operating room after tooth extraction by OMFS to perform a bilateral nasal endoscopy, right maxillary antrostomy, right total ethmoidectomy, right sphenoidotomy, and right frontal sinusotomy with balloon dilation. His sinus cultures were positive for 2+ microaerophilic streptococci. He was treated with clindamycin 900mg IV every eight hours for a total of three days and discharged on oral clindamycin 450mg every eight hours to complete 14 total days on antibiotics.

His WBC count decreased from 22.7 * 10^9^ to 7.7 * 10^9^ after IV antibiotics and surgical interventions. Ophthalmologic consultation reported mildly elevated intraocular pressures (IOPs) of the right eye between 22-26, both before and after ENT's intervention. He remained afebrile throughout and no complications were documented. He was discharged on day 4 in stable condition.

## Discussion

The best way to differentiate postseptal cellulitis from preseptal cellulitis is by its classic clinical features which include painful eye movements, proptosis, and ophthalmoplegia, and by either obtaining a CT scan or magnetic resonance imaging (MRI). Conjunctival swelling (chemosis), fever, and peripheral leukocytosis with a left shift are more commonly seen in a patient with postseptal cellulitis, but can also be present in patients with preseptal cellulitis [1,2].

Preseptal cellulitis of the orbit is much more common than postseptal cellulitis, and both of these conditions are more common in children than adults. The most common cause of postseptal cellulitis is bacterial rhinosinusitis. Approximately 86-98% of cases of postseptal cellulitis have coexisting rhinosinusitis [2-5]. Other potential causes include surgery of the eye or eyelids, peribulbar anesthesia, orbital trauma with a fracture or a foreign body, dacryocystitis, tooth infection, otitis media or an infected mucocele that erodes into the orbit [6-19].

The most common pathogens associated with postseptal cellulitis are Staphylococcus aureus and streptococci [2-4, 18]. However, some studies have documented the presence of polymicrobial infection including aerobic and anaerobic bacteria and/or fungi, especially Mucorales and Aspergillus species [19]. Fungal infections should always be considered, especially in patients who are immunocompromised.

Most cases of postseptal cellulitis can be managed with oral or IV antibiotics. However, it is important to always evaluate for potential complications such as the development of a subperiosteal abscess, an orbital abscess, loss of vision, destructive sinus disease, and intracranial extension [20], as such was the case with our patient. Patients with extension of their infection should be followed by the ophthalmology, ENT and OMFS services in the event that surgery becomes indicated for any of the listed complications. The most common complications of postseptal cellulitis are subperiosteal abscess and orbital abscess formation. Both of which can develop very quickly.

The current case describes many of the complications of periorbital cellulitis described in the literature, for which surgical intervention was required. This case highlights the importance of a thorough physical exam and review of imaging studies.

## Conclusions

Patients with suspected orbital cellulitis should be monitored very closely, and have their visual acuity and their pupillary light reflex assessed daily. An absent or sluggish pupillary light reflex could indicate optic nerve involvement. Other signs that would indicate the formation of a subperiosteal or orbital abscess are a proptotic eye or elevated intraocular pressures. A contrast CT scan of the orbits and paranasal sinuses would be useful in detecting a subperiosteal or an orbital abscess.
